# Immune evader cancer stem cells direct the perspective approaches to cancer immunotherapy

**DOI:** 10.1186/s13287-022-02829-9

**Published:** 2022-04-08

**Authors:** Hassan Dianat-Moghadam, Amir Mahari, Reza Salahlou, Mostafa Khalili, Mehdi Azizi, Hadi Sadeghzadeh

**Affiliations:** 1 Department of Genetics and Molecular Biology, School of Medicine, Isfahan University of Medical Sciences, Isfahan, Iran; 2 Department of Biomedical Engineering, University of Arkansas, Fayetteville, AR USA; 3 Biotechnology Research Center, Tabriz University of Medical Sciences, Tabriz, Iran; 4 Blood Transfusion Research Center, High Institute for Research and Education in Transfusion Medicine, Tehran, Iran; 5 Department of Tissue Engineering and Biomaterials, School of Advanced Medical Sciences and Technologies, Hamadan University of Medical Sciences, Hamadan, Iran; 6 Department of Tissue Engineering, Faculty of Advanced Medical Sciences, Tabriz University of Medical Sciences, Tabriz, Iran

**Keywords:** Tumor microenvironment, Cancer stem cells, Immune evasion, CAR T cell, CAR NK cells, Nano-immunotherapy

## Abstract

Exploration of tumor immunity leads to the development of immune checkpoint inhibitors and cell-based immunotherapies which improve the clinical outcomes in several tumor types. However, the poor clinical efficacy of these treatments observed for other tumors could be attributed to the inherent complex tumor microenvironment (TME), cellular heterogeneity, and stemness driven by cancer stem cells (CSCs). CSC-specific characteristics provide the bulk tumor surveillance and resistance to entire eradication upon conventional therapies. CSCs-immune cells crosstalk creates an immunosuppressive TME that reshapes the stemness in tumor cells, resulting in tumor formation and progression. Thus, identifying the immunological features of CSCs could introduce the therapeutic targets with powerful antitumor responses. In this review, we summarized the role of immune cells providing CSCs to evade tumor immunity, and then discussed the intrinsic mechanisms represented by CSCs to promote tumors’ resistance to immunotherapies. Then, we outlined potent immunotherapeutic interventions followed by a perspective outlook on the use of nanomedicine-based drug delivery systems for controlled modulation of the immune system.

## Introduction

Medical scientists have developed diversified treatments for cancer therapy; nonetheless, cancer mortality remains one of the biggest problems for humans worldwide. Tumor immunogenicity is induced by the antitumor activities of dendritic cells (DCs) and macrophages which activate the tumor-associated antigens (TAAs)-specific T cells resulting in the production of necessary cytokines and engagement of γδ T cells, natural killer (NK) cells, and B cells, all providing antitumor cytotoxicity [1]. Inspired by these findings and aimed to improve the outcomes of cancers therapy, scientists focused on the development of immunotherapies based on antibodies, cancer vaccines, soluble cytokines, and immune cell-based therapy [2, 3].

However, the genetic and cellular heterogeneity of the surrounding tumor microenvironment (TME) complicated the clinical efficacy of those treatments, which requires further understanding of the multiple aspects of tumor biology [4]. Along with the genetic and epigenetic instabilities, the nongenetic intra-tumor heterogeneity and plasticity conduct therapy failure due to the presence of the rare population of cancerous cells known as cancer stem cells (CSCs) or cancer-initiating cells (CICs) [5, 6]. The self-renewal abilities of CSCs and the reversible differentiation of non-CSCs into CSCs all indicate their malignant characteristics to be actors of tumor recurrence, metastasis, and resistance to existing therapies [6]. CSCs/CICs are identified by dynamic states of metabolome and cellular differentiation [7], their surface proteins (e.g., CD24, CD133, CD44, and LGR5), and intracellular molecular signature including aldehyde dehydrogenases (ALDHs) activity, overactivation of drug carriers (i.e., ABC transporters), and signaling pathways (e.g., Hedgehog, Notch, Wnt/β-catenin) [5, 6]. The protected niche of CSCs is provided by overactivation of anti-apoptotic effectors, entering in dormancy state, activating autophagy during metabolic stress, and enhancing DNA damage response (DDR) to drugs targeting DNA synthesis or structure [6].

The tumor site-infiltrated immune cells are expected to target and eradicate cancer cells. However, the failure of immune responses and switching from immunosurveillance to tumor surveillance occurs due to increased niche complexity and emerged immunosuppressive TME is produced by CSCs alone or in cooperation with other non-cancerous cells including tumor-supportive immune cells [8, 9]. Reprogramming of TME as well as improving the efficacy of current immunotherapies or expanding the immunotherapeutic options demands clarifying the cellular and molecular interactions between CSCs and the immune system, as presented in this review.

## CSCs negotiate with tumor immunity

### CSCs and dendritic cells

**Fig. 1:**
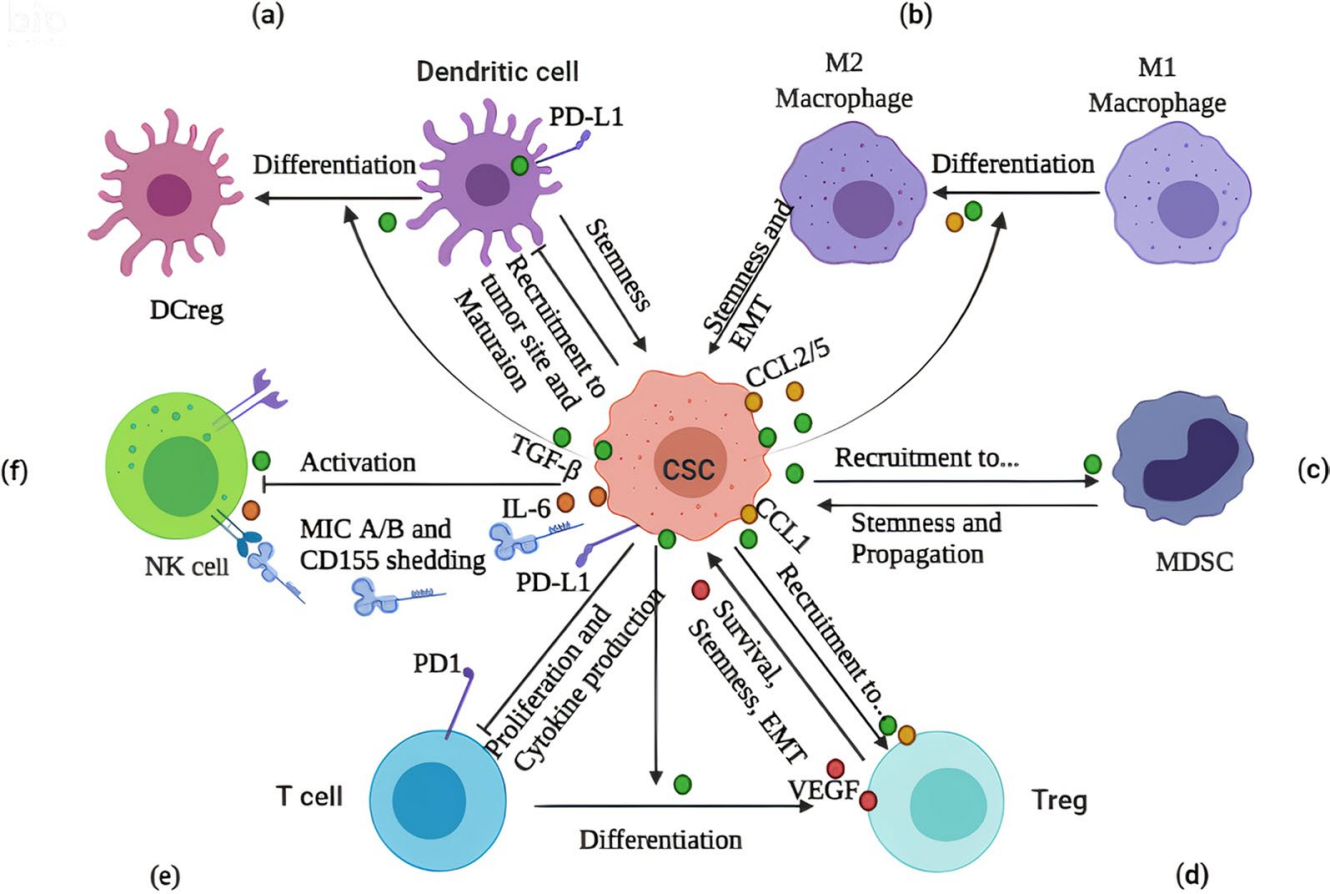
Interaction between immune cells and CSCs in the immunosuppressive tumor microenvironment. **a** CSCs impair recruiting and maturation of DCs in tumor sites or use TGF-β signaling to induce DC differentiation into DCregs. **b** CSCs-derived TGF-β/CCLs induce the differentiation of M1 macrophage to M2 phenotype ones vs. M2 macrophages induce EMT and stemness in CSCs. **c** CSCs-derived TGF-β recruits MDSCs in tumor sites and reciprocally, MDSCs support CSCs stemness and propagation. **d** CSCs-derived TGF-β also recruits Tregs in tumor sites and reciprocally, Tregs-derived VEGF induces angiogenesis supporting CSCs survival and EMT. **e** PD-L1 + CSCs suppress T cells activation or induce their differentiation into Tregs-supporting tumors. **f** CSCs-derived IL-6 and TGF-β downregulate NK-activating receptors, and also, CSCs suppress NK cells activation through re-expression of MHC-II or shedding of MIC A/B and CD155-suppressing activating receptors

Mature dendritic cells (DCs) are major antigen-presenting cells (APCs) which present TAAs on MHC-I molecules plus overexpress costimulatory molecules resulting in activate antigen-specific T cells-directed immune responses [1]. However, TME-associated CSCs or stromal cells interfere with DCs recruitment to the tumor site, impair their maturation, and promote their differentiation into immunoregulatory DCs (DCregs) with immunosuppressive behaviors [4] (Fig. 1a). CSCs secrete transforming growth factor-β (TGF-β) or upregulate its signaling which interferes with DCs antitumor responses by several mechanisms including (i) the reduction of mature DCs, or enhancement of the populations of tolerogenic DCs or DCregs [10], (ii) downregulation of MHC class II and DCs-costimulatory molecules (i.e., CD80 and CD86) [11], (iii) inhibition of CD103^+^DCs recruiting to the tumor site in Wnt/β-catenin-dependent pathway [12], and (iv) the development of PD-L^+^ DC impairing CD8^+^T cell activity or rising the resistance to anti-PD1 treatment [13]. In renal cell carcinomas, the CD105^+^ CSCs-derived extracellular vesicles (EVs) carry a soluble type of MHC-I, HLA-G (sHLA-G), which act as a ligand for immunoglobulin-like transcript (ILT) inhibitory receptors and thus impair monocyte differentiation into mature DCs through IL-6–STAT3 signaling pathway [14, 15]. In contrast, tumorigenic DCs contribute to the maintenance of CSCs, whereby the interaction between CXCL12 of DCregs and CXCR4 of glioma CSCs (GSCs) trigger signaling pathway-supporting GSCs niche homing and survival [16]. In addition, the CXCL1-expressing DCs induce stemness signaling of NANOG, OCT4, SOX2, and MYC in CD133^+^ colon cancer cells with high metastatic ability [17].

### CSCs and macrophages

The interaction between normal tissue-resident stem cells (SCs) and macrophages is essential for organ development, tissue homeostasis, and regenerative medicine [18]. This interaction also has been found in cancerous tissues but in different pathways where tumor-associated macrophages (TAMs) support CSCs maintenance, and their cooperation provides the immunosuppressive TME [6] (Fig. 1b). CSCs niche enriching of C–C motif chemokine 2/5 (CCL2/5), interleukins (ILs), extracellular matrix (ECM) protein periostin, TGF-β, and colony-stimulating factor1 (CSF1) facilize the recruitment and polarization of protumorigenic macrophage into immunosuppressive M2 or TAM phenotypes [19, 20]. For example, GSCs-expressing periostin binds to integrin αvβ3 and thus recruits macrophages from the peripheral blood to the brain [20], where TAMs support CSCs activity and survival through a series of cell-intrinsic and cell-extrinsic molecular mechanisms. TAM-derived TGF-β induces NF-κB (nuclear factor-κB), AKT, and STAT3 (signal transducer and activator of transcription 3) signaling pathways in CSCs and thus, sustains their self-renewal and stemness state [21]. TGF-β also produces the EpCAM^+^ CSC-like cells through induction of epithelial to mesenchymal transition (EMT) program and these cells, in turn, promote hepatocellular carcinoma (HCC) invasion and metastasis [21].

Alternatively, TAMs induce the overexpression of CD47 ligand on pancreatic CSCs [22], HCC CSCs [23], and leukemia stem cells [24], which bind to signal-regulatory protein α (SIRPα) on phagocyte cells and thus protect them from cell-mediated phagocytosis. Regarding adaptive immune responses, TAMs stimulate immune checkpoints expression such as programmed cell death protein-1 (PD-1) and its ligand, PD-L1 in T cells and CSCs, respectively, and thus impair T-cell cytotoxic activity [25]. Overall, CSC–TAM communication induces immunosuppressive TME which supports CSCs survival and complicates the bulk tumor eradications upon immunotherapy.

### CSCs and myeloid-derived suppressor cells

Human myeloid-derived suppressor cells (MDSCs) are myeloid-originated progenitor cells that are classified into two subsets; monocytic MDSCs (M-MDSCs) and polymorphonuclear MDSCs (PMN-MDSCs). MDSCs are defined by CD11b^+^CD14^–^CD33^+^ and they secrete cytokines and chemokines in TME providing immunosuppressive niche and thus, reducing the efficacy of immunotherapy [6]. Mammalian target of rapamycin (mTOR) signaling in CSCs mediates the enhancement of colony-stimulating factor (CSF) that supports MDSCs infiltration and accumulation in tumor site [26]. During melanoma formation, TGFβ1-overactivated CD133^+^CSCs have been shown to recruit the immunosuppressive MDSCs in the tumor site [27] (Fig. 1c). Moreover, leukemic stem cells (LSCs)-expressing T cell immunoglobulin mucin-3 (TIM-3)/Galectin 9 pathway expands the MDSCs population and also, differentiates them into TAMs leading to impairment of T cell response [28].

Reciprocally, MDSCs contribute to the stemness and survival of cancer cells via multiple mechanisms including (i) the modulation of RNA interference such as upregulation of piRNA-823 to induce NANOG, OCT4, and SOX2 expression [29], (ii) the epigenetic regulation such as secretion of exosomal S100A9 to enhance STAT3/NF-κB phosphorylation which promotes the stemness of cancer cells [30], and (iii) the production of tumor-supportive simulators such prostaglandin E2 (PEG-E2) which expand the ALDH^+^ CSCs population in uterine cervical cancer [31]. These findings revealed that the MDSCs-CSCs interaction reshapes the stemness in tumor cells resulting in tumor formation and progression.

### CSCs and regulatory T cells

Regulatory T cells (Tregs) are a T cells-originated subset that has been shown to crosstalk with CSCs in several mechanisms to promote an immunosuppressive TME [6] (Fig. 1d). CSCs-derived PD-L1 and TGF-β mediate Tregs infiltration in the glioblastoma site and their tumor-supportive effects are related to poor survival and promotion of cancer stemness [32]. In another pathway, SOX-expressing CSCs produce CCL1which recruits Tregs to the TME [33], and after that, Tregs produce TGF-β and IL-17 to promote self-renewal ability, stem cell markers, and EMT toward tumor progression and invasion [34, 35]. STAT3 signaling supports gastric CSCs maintenance by inhibiting cell apoptosis. They also protect CSCs from T cell killing by the differentiation of uncommitted CD4^+^T cells into Tregs [36]. In hypoxic TME, Tregs secrete vascular endothelial growth factor (VEGF), as a mediator of angiogenesis supporting CSCs survival, stemness, and self-renewal [37]. Moreover, Tregs-derived cyclooxygenase 2 (COX-2) suppresses effector T cells in the PEG-E2-dependant mechanism, confirming that the CSC niche-associated Tregs promotes immune evasion that can potentially contribute to failure in cancer immunotherapy [38].

### CSCs and T cells surveillance

Normally, T cells recognize overexpressed TAAs in the form of MHC/peptide complexes on the APCs surface. However, CSCs-TAAs have shown low immunogenicity which is related to their sharing with normal tissues and the presence of tolerogenic T lymphocytes recognizing these antigens. In addition, CSCs escape immunosurveillance and recognition by antitumor immunity through (i) suppressing T-cell activation [39], (ii) the downregulation of TAAs such melanoma CSCs [40], (iii) the modulation of MHC-1 expression at the transcriptional and posttranscriptional levels, or expression of the mutated MHC-1 [41, 42], and (iv) the overexpression of immune checkpoints such PD-L1 [43] (Fig. 1e). The downregulation of MHC-1 expression also affects CD8^+^ T cells activation to target the CSC-neoantigens generating through tumor-specific somatic mutations [44]. EMT/β-catenin signaling engages the ER-associated N-glycosyltransferases, STT3, which regulates the immune checkpoint PD-L1 glycosylation and stabilization and thus, develops enriched PD-L1 expression in CSCs, providing additional benefits to CSCs evade T cells immunosurveillance [45]. In the hypoxic niche, HIF-1-expressing CSCs induce VEGF expression that promotes the expression of PD-L1 and T cells inhibitory receptor TIM-3 impairing their function [46].

CSCs also express other immune checkpoint molecules such as CD276, CTLA4 (cytotoxic T lymphocyte antigen-4), and Galectin-3 (Gal-3) as an ECM glycan-binding protein. During HNCC development and metastasis, the CD276^high^ CSCs are located in the front of invasive tumors and provide a physical shield against the infiltration of CD8^+^ T cells to support immune evasion [47]. TGFβ develops CD80 ligand-expressing CSCs that engage Tregs and interact with CTLA4 on T cells and thus mediate the resistance to cytotoxic T cell activity or T cell-based immunotherapy [48]. In another mechanism, prostate CSCs overexpress cytoplasmic Gal-3 which inhibits T cells receptor (TCR) clustering, dampens the T cell proliferation and cytokine production, and therefore, protects CSCs against cytotoxic T cells [49].

Thanks to CSC plasticity, another strategy for evading CSCs from the immunosurveillance is developing the slow-cycling or quiescent CSCs supporting long-term survival and maintaining tumor-initiating potential in these cells [6]. Quiescent SCs protect from T cell detection and killing through downregulating the NLRC5 (NOD-, LRR- and CARD-containing 5) transactivator belonging to MHC class I-dependent immune responses [50].

### CSCs and NK cells surveillance

NK cells expressing activator NKG2D ligands, NKG2DL, or activating receptors (i.e., NKp30 and NKp44) are able to kill MHC-1-negative CSCs in an APC-independent manner [51]. Compared with non-CSCs, the NK cell-mediated cell lysis has been also reported in MHC-1-negative colon CD133^+^CD44^+^CSCs/CICs expressing NKG2DL [52], and MHC-1-negative ovarian CD24^+^CSCs expressing NKp30 and NKp44 [53]. However, CSCs in patients with ovarian carcinoma [54] and renal carcinoma [55] have been shown to upregulate MHC-I molecules, making them less sensitive to NK cell-mediated cell lysis (Fig. 1f). NK cells-inhibitory ligands KIR2DL4 and NKG2A interact with HLA-G and HLA-E on CSCs and directly inhibits NK-cell activation [56]. Moreover, down-modulation of NKG2DL on CSCs affects the immunogenic profile of these cells to activate NK cells responses. For example, SOX2/SOX9-expressing CSCs produce Wnt inhibitor DKK1 and raise dormant CSCs (or LCC, latency competent cancer cells) with autonomous downregulation of NKG2DL that protect them from NK cell-mediated clearance [57]. The depletion of NK cells leads to loss of this developmental stage in latency phase or metastatic dormancy of CSCs which is characterized as potent refractory to NK cells-based immunotherapies responsible for tumor relapse [58]. Therefore, understanding the potentiate mechanisms that drive NK cells mediated recognition and elimination of CSCs may pave the way for a new generation of anti-CSC targeted immunotherapies.

## Immunotherapies targeting CSCs

Although clinical evidence has demonstrated the efficiency of anti-CTLA-4 and anti-PD-1/PD-L1 antibodies in targeting bulk tumor cells, a large number of patients still do not derive benefit from them. A deeper understanding of the unique interactions between CSCs and the immune system can contribute to developing efficient immunotherapies capable of targeting pathways responsible for CSCs acquiring immune evasion as summarized below.

### NK cells immunotherapy targeting CSCs

Resistant CSCs overexpress metabolic vulnerabilities that can be targeted by immunomodulators or drugs directed to CSCs-associated pathways. MHC-Ib-expressing CSCs are vulnerable to NK cell-mediated cytotoxicity in an NKG2DL-dependent mechanism [59], whereby breast CSCs shed MHC-Ib to promote an immune evasive state [60]. Furthermore, CD34^±^ AML stem cells have shown high expression of poly-ADP-ribose polymerase 1 (PARP1) which is responsible for the repression of NKG2DL. This subset of NKG2DL-negative cells mediates evasion from NK cells clearance, and thus inducing genetic or pharmacologic inhibition of PARP1 leading to re-expression of NKG2DLs on the AML stem cells surface and re-sensitizing them to NK cells [61].

The low infiltration of injected NK cells in the solid tumors may contribute to the development of targeting circulating CSCs in the bloodstream [62]. The melanoma CCR7^+^CSCs exhibited upregulation of NKp30/NKp46 ligands and downregulation of MHC-I molecules making them sensitive to NK-cell-mediated cell lysis [63]. The interaction between the NKG2D receptor and CSCs-overexpressing MHC-I polypeptide-related sequence A/B (MIC A/B) ligands enhanced their killing by NK cells [59]. Additionally, transplanted NK cells with short half-life properties overcome the autoimmunity problem. They do not also induce overproduction of pro-inflammatory cytokines [62]; the benefits that potentiate allogenic or autologous NK cells and engineered chimeric antigen receptor (CAR)-NK cells to eliminate CSCs with high-affinity targeting. NK-resistant GSCs could be re-sensitized using cytokine-induced killer (CIK) cells such lectin/IL2-activated NK cells but in optimized doses to protect against expansion of immunosuppressive Tregs [64]. Similar to NK cells, CIKs identified with MHC-independent antitumor activity kill CSCs-expressing MIC A/B via NKG2D recognition [65]. Coupling of the tumor-killing capacity and CAR engineering directed studies can lead to developing CAR CIKs specific for CSCs antigens such as CD44v6, 5T4, and CSPG4 [66, 67]. These CAR CIK cells could efficiently eliminate CSCs in vitro and in vivo, thus introducing a cancer immunotherapy option that needs further evaluation in more preclinical studies in synergism with other therapeutic strategies.

Regarding CAR NK cells, epidermal growth factor receptor (EGFR) as breast CSCs-TAA was eliminated in vivo in mice model using EGFR-CAR NK-92 cells, and its therapeutic efficacy was improved when combined with FDA-approved oncolytic herpes simplex virus (oHSV) [68]. Metastatic CSCs overexpress EpCAM antigen [69], and the administration of EpCAM-CAR NK-92 cell-coexpressing IL-15 has been shown to induce CAR NK cells proliferation with high selective activity against EpCAM-positive carcinoma cells in vivo [70].

Nonetheless, the downregulation of TAA in CSCs [40] and expression of CSC markers in normal cells [4] limited their targeting efficacy by T or NK cells that could be substituted using embryonic stemness markers such as octamer-binding transcription factor 4 (OCT4) and sex-determining region Y-box 2 (SOX2). These marker proteins are expressed by CSCs but not normal adult cells [4], and the detection of anti-SOX2 antibodies in lung tumor tissue [71] suggests the immune-editing targeting stemness transcription factors in human tumors. Considering dormant CSCs, the selectively re-activating NK cell ligands in quiescent metastatic cells might trigger the immunologic elimination of latent metastasis [57].

### T cells immunotherapy targeting CSCs

**Fig. 2:**
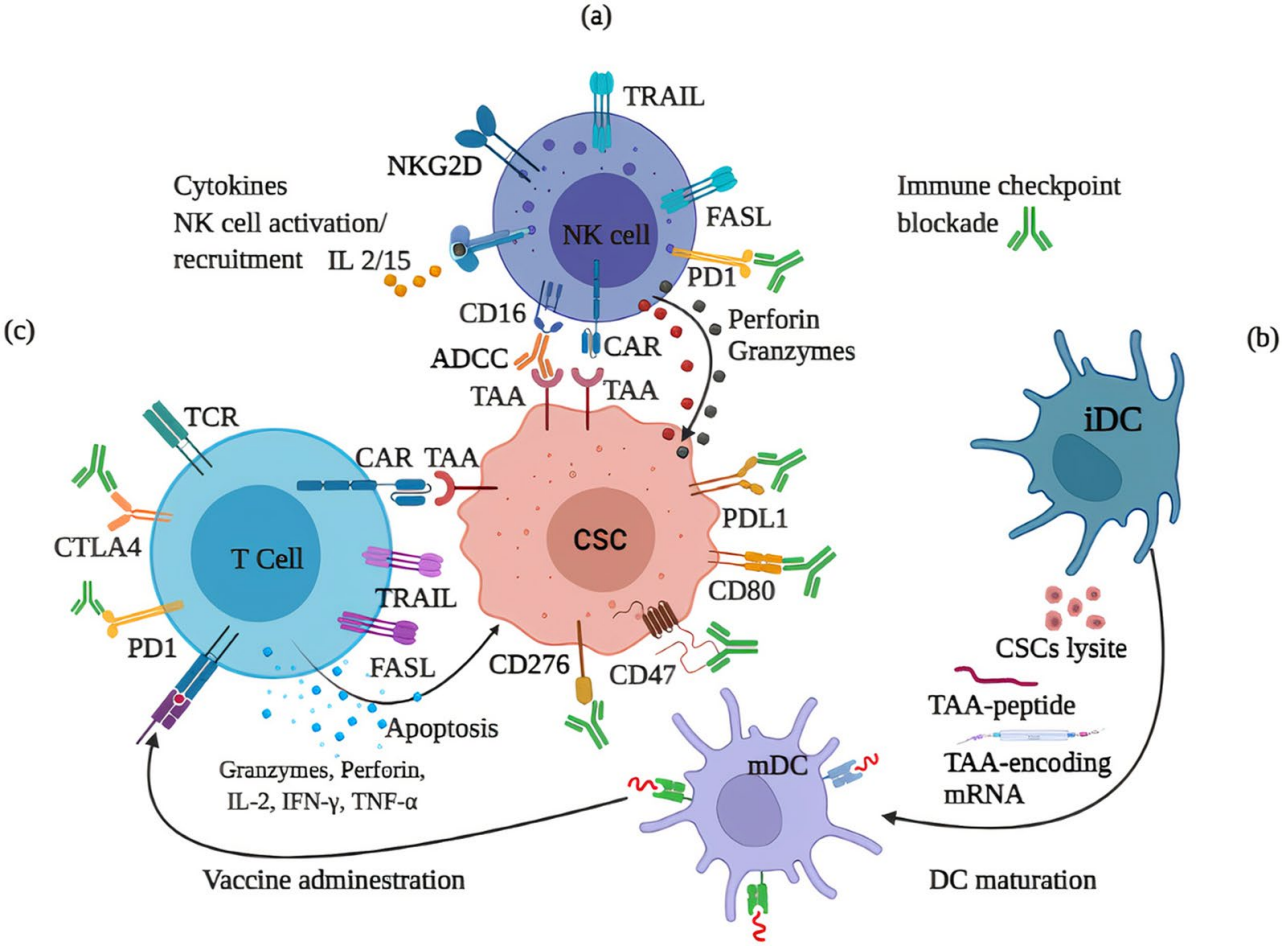
Immunotherapies targeting CSCs. **a** Administrated NK cells or CAR NK cells target TAAs on CSCs and induce cytolysis and apoptosis via perforin/granzymes and TRAIL/FASL, respectively. Moreover, therapeutic antibodies target CD16 and TAA to mediate the ADCC process. **b** Ex vivo maturation of DCs exposed to CSCs-lysate/TAAs/peptides produce a vaccine that after administration arm the cytotoxic T cells in an MHC-1-TCR-dependent manner for targeting specific CSCs. **c** Administrated T cells or CAR T cells induce cytolysis and apoptosis in CSCs. Antibodies targeting immune checkpoint molecules such as PD1/PDL1, CD276, and CTLA4 could improve the anticancer immune responses. Anti-CD47 antibody sensitizes CSCs to cell-mediated phagocytosis. ADCC, antibody-dependent cellular cytotoxicity; FASL, FAS ligand; iDC, immature DC, mDC, mature DC; TRAIL, TNF-related apoptosis-inducing ligand

The DCs-based vaccines have been developed as a promising and effective strategy to facilitate CSC eradication, and in two examples, DCs loaded with CSCs-lysate [72] or CSCs-derived exosomes [73] have shown promising preclinical immunotherapeutic outcomes. However, defects in MHC-I and the presence of tolerogenic T cells recognizing TAAs result in the low immunogenicity of these antigens. Further, the upregulation of PD-L1 exerts immunosuppressive effect leading to low efficacy DCs vaccine [1]. These results advocate the use of the DCs vaccine in combination with CIKs and chemo agents and checkpoint inhibitors (Fig. 2b). Co-administration of CSCs antigens-loaded DCs and CIKs results in the elimination of liver CSCs and tumor growth inhibition in nude mice models [74]. Most recently, co-treatment with CSC-DC-based vaccine and low doses of cisplatin illustrated effective antitumor responses and prolonged survival rate of solid Ehrlich carcinoma (SEC)-bearing mice model [75]. In the niche of thyroid cancer, the neurons-secreted acetylcholine (ACh) promotes the self-renewal and immune escape of CSCs by promoting CD133-Akt phosphorylation which subsequently induces PD-L1 expression. Moreover, the administration of 4-DAMP, an ACh receptor M3R antagonist, re-sensitizes CD133^+^ CSCs to CD8^+^ T cell-mediated cytotoxic activities and inhibits the cancer growth in vivo [76].

Regarding immune checkpoints molecules (Fig. 2), the combinatorial of bevacizumab as VEGF inhibitor and PD-L1 inhibitor atezolizumab showed promising outcomes in advanced HCC cases as approved by FDA for HCC therapy [77]. The antibodies that target CTLA-4 can render stem cell susceptible to treatment [48]. A recent study has also shown that target CD276 blockade uses an antibody to eliminate CSCs in HNSCC and inhibit metastasis [47]. However, targeting immune checkpoints using antibody monotherapy does not achieve the same efficacy, which highlights these therapies may be applied in combination with other immunotherapies. To this end, the results from preclinical animal models demonstrated that anti-PD-L1 etoposide synergizes with Tim-3 blockade therapy [45]. These findings open the field for combining CSC-loaded DC vaccine with other conventional therapies such as targeting the negative immune regulation as an effective strategy to improve anticancer immune responses.

The T cells suppressor and CSCs supportive effects of Tregs-derived COX-2 and its product, PEG-E2 [38], could be reversed using the optimized concentration of interferon-gamma (IFN-γ) [78]. IFN-γ can also target another metabolic stress-associated response in CSCs such as autophagy, as the process supporting CSC survival and failure treatment [79], confirming that the functions of CSC metabolisms must be considered in cancer immunotherapy.

Regarding adoptive cell therapy (ACT), IFN-γ has been shown to upregulate the MHC-I molecules in HLA class I-defective CSCs and thus, make sensitive their neoantigen reorganization by T cells to efficiently target CSCs populations [44]. Further, the transplantation of γδ T cells upregulated MHC- I and CD54/ICAM-1 on breast CSCs and thus increased their susceptibility to CD8^+^ T-cells killing in an MHC-restricted manner in vivo [80]. Similar to CAR NK cells, there are several CAR T cells have been developed and tested in preclinical and clinical studies. AC133-CAR T cells targeting GSCs in glioblastoma inhibit the growth of orthotopic xenografts initiated from GSCs, and the low survival of CAR T cells for targeting the invasive GSCs could be addressed using an additional CAR with an inhibitory receptor with specificity for normal tissue antigens [81]. Ephrin type-A receptor 2 (EphA2) CAR T cells have been developed to target the EphA2^+^ U373 glioma xenograft mouse model and the results indicated that CARs with a short spacer had significantly greater antitumor activity, which could be augmented by transgenic expression of cytokines to counteract the immunosuppressive TME [82]. Promising preclinical results encourage testing a phase I clinical study to evaluate the therapeutic effects of CD133-directed CAR T cells in 23 patients with advanced HCC (Clinical Trials ID NO: NCT02541370). The data indicated that 21 out of 23 patients had not developed detectable de novo lesions after CD133 CAR T cells infusions and revealed that a longer period of disease stability demanded CD133 CAR T repeat infusion [83]. However, the expression of CD133 on the bile duct endothelium led to severe toxicity in some patients, highlighting that CD133 CAR T cell therapy must be approached with great caution in patients with a background of bile duct stenosis [83].

Immunosuppressive TME contributes to the development of immunotherapeutic interventions for the eradication of malignant diseases [6]. Targeting the CD47/SIRPα, Hu5F9-G4 antibody has been used in combination with Azacitidine, and the results have shown promising effects in leukemia stem cells (NCT03248479). The chondroitin sulfate proteoglycan 4 (CSPG4) is upregulated in CSCs by hypoxic TME and immune targeting of CSPG4 using specific antibody and CSPG4 CAR T cells have been shown to eliminate CSPG4-positive CSCs/neurospheres in immunodeficient mice and in vitro, respectively [84]. Tumor endothelial marker 8 (TEM8) is an integrin-like cell surface protein that induces angiogenesis supporting tumor invasion and also overexpresses in triple-negative breast cancers (TNBC)-associated CSCs [85]. On other hand, the CSCs stemness is supported by angiogenesis. TEM8 CAR T cells TNBC was designed against breast CSCs and tumor-associated vasculature resulted in killing TNBC tumor cells, the inhibition of mammosphere formation, impairing tumor neovasculature formation, and inducing the regression of established PDXs in local and metastatic tumors [85]. A clinical study combining anti-EGFR CART cells and anti-CD133 CAR T cells indicated a potential treatment for patients with advanced cholangiocarcinoma [86]. Following the above-mentioned trail (NCT02541370), the expression of CD133 on circulating endothelial progenitor cells (EPCs), as actors of angiogenesis and neovascularization in advanced HCC, another trial study was conducted to target CD133 on both of CSCs and EPCs and then analyze the changes in circulating multiple plasma molecules in response to CD133 CAR T cells (NCT02541370). Outcomes have shown that after cell infusion the baseline levels of VEGF and stromal cell-derived factor (SDF)-1 significantly increased, whereas soluble VEGF receptor 2 (sVEGFR2) and platelet-derived growth factor (PDGF) levels and EPCs count significantly decreased [87]. Further, an increase in circulating IFN-γ indicated CD133 CAR T cells is a more favorable antitumor activity that could be achieved upon combinatorial therapy of CAR T cells angiogenic inhibitors [87].

Encouraging results directed CAR-T cell therapy against CSCs in other clinical studies (Fig. 2c). However, the raised off-tumor toxicity and autoimmunity challenged the safety and clinical responses of CAR T cells which could be considered through (i) developing in vivo controllable ones by encoding suicide genes in CAR T cells, (ii) increasing tumor specificity CAR T cells by using bispecific CARs, (iii) using switchable molecules that improve unfavorable effects of the CAR T cells activity, and (iv) combine CAR T cells with checkpoint antibodies or antiangiogenic to provide a more favorable immune microenvironment for antitumor activity [1, 6, 87, 88].

### Nano-immunotherapy targeting CSCs

**Fig. 3:**
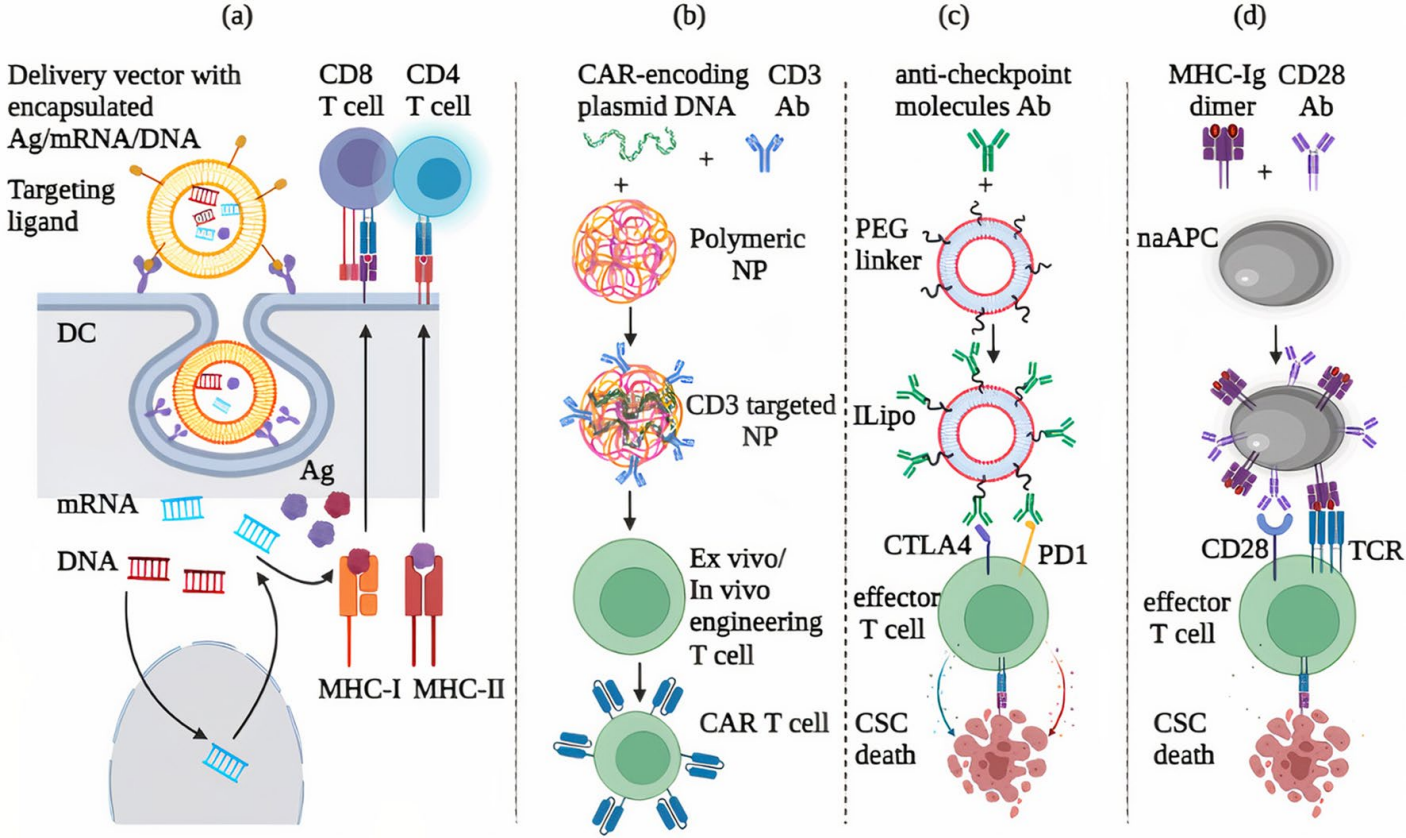
Prospective nano-immunotherapy approaches to target CSCs. **a** The nucleic acids-based vaccine (mRNA or DNA) and CSCs-specific neoantigens are formulated into the nanoparticle displaying a DCs-targeting ligand to engage DCs followed by activation of CD4/8T cells in vivo. **b** Plasmid-encoding CAR against various CSC proteins can be loaded in CD3 targeted polymeric nanoparticles that genetically reprogramme T cells ex vivo or in vivo and thus T cells can be armed specificity to CSCs antigen of choice. **c** Owing to improving the pharmacokinetics of the immunomodulators and lowering their systemic exposure, the multiples of anti-PD1 and anti-CTLA4 antibodies can be bioconjugated to the surface of PEGylated liposomes to target T cells in vivo and promote their antitumor immune responses. **d** naAPC can generate coupling of co-stimulatory anti-CD28 antibody and MHC-1-Ig dimer on the surface of magnetic nanoparticles which induce TCR clustering and expand and activate the tumor-infiltrated T cells efficiently in vivo. Ag, antigen; ILipo, immunoliposomes; PEG, polyethylene glycol; naAPC, nano artificial antigen-presenting cell

Immunotherapies could be combined with CSCs-targeted nano agents/carries with potentials to both debulk the original tumor and eradicate the emerging resistant CSCs [6]. Indeed, nanoparticles (NPs) have been patented to provide a protective system to package the desired doses of immune agents (i.e., cancer vaccines, CAR T, and checkpoint inhibitors) with safe delivery enabling the improvement of efficacy of cancer treatment [1] (Fig. 3).

Without the use of toxic transfection reagents, the co-delivery of both antigen and adjuvant in such nanovaccines leads to targeting the same APCs maximizing the benefits of the intended vaccinal effect. Nanovaccines provide the intranuclear and intracellular delivery of DNA and mRNA vaccines, respectively (Fig. 3a) while improving their immunogenicity and stability against degradation by nucleases [89]. Neoantigens are unique to each patient and can be used to produce personalized cancer vaccines and are ideal for targeting MHC-defective CSCs in heterogeneous cancers [44]. Nonetheless, the systemic application of this type of vaccine is limited due to reduced stability and raised off-target adverse effects which could be addressed using nano delivery systems protecting several neoantigens within one platform [89] (Fig. 3a).

Lymph nodes (LNs) are the micrometastasis sites of migrating CSCs first reenter hereafter circulating, and further, CSCs induce lymphangiogenesis-supporting tumor metastasis [90]. On the other hand, the low infiltration of administrated free immuno agents into LNs restricted their efficacy, which leads to improved next-generation vaccines enabling the co-delivery of antigen and adjuvant to lymph nodes and promoting robust and sustained antigen presentation by APCs. To this end, a lipid-based NP, RNA-lipoplexes (RNA-LPX), was designed to target anionic mRNA in DCs [91]. RNA-LPX is concentrated in lymphoid organs, induce IFNα release by activated DCs, and elicit the memory and antigen-specific T-cell responses in murine models and even patients with melanoma (NCT02410733) [91]. In another study, the high-density lipoprotein (HDL)-mimicking nanodisks containing neoantigen, 5C-phosphate-G-3(CpG) motif (as Toll-like receptor-9 agonist), and cholesterol (as an enhancer of in vivo trafficking) was developed [92]. Vaccination with these nanodisks has been demonstrated to significantly improve co-delivery of antigen/adjuvant to lymphoid organs, induce the maturation of DCs and elicit the neoantigen-specific CTLs responses results in the killing of target melanoma cells in peripheral tissues, and established once when combined with anti-PD-1 and anti-CTLA4 therapy [92].

Primary CSCs are located in the center of primary solid tumors with restricted cytotoxic lymphocyte infiltration [62, 93], and delivery platforms can employ the matrix metalloproteinases (MMPs) to overcome extracellular barriers and increase immune agents’ exposure at this particular site [94]. An alternative way for targeting CSCs directly is the use of nanoparticles. Since immune cells are present in the circulating blood with high frequency [62], nanoparticles can directly target endogenous T cells in the bloodstream for improving the traditional adoptive CAR T cell therapy. For example, one study designed CD3-targeted polymer NPs encapsulating tumor CAR-encoding DNA which efficiently targeted and delivered DNA into the nuclei of T cells and programmed them in situ in mouse models of B cell lymphoblastic leukemia resulting in long-term disease remission [95] (Fig. 3b). This is a cost-saving and potentially safer strategy that provides the engineering T cells in situ without the requirement of ex vivo laborious processes.

Immunomodulatory antibodies target CSCs expressing CTLA-4 or CD276 promoting robust antitumor immune responses, but the induction of off-tissue effects is reported in clinical settings [47, 48]. To provide safe delivery of these antibodies, targeting endogenous T cells using nanoparticles conjugated with immunomodulatory antibodies allowed tumor-infiltrating T cells to take up nanoparticles (Fig. 3c). An alternative to delivering nanomaterials to T cells is the local injection of the biodegradable microspheres composed of poly(d,l lactic-co-hydroxymethylglycolic acid) (pLHMGA)) that were utilized to present a controlled release system of anti-CD40 (stimulating DCs) and anti-CTLA-4 antibodies in colon carcinoma tumor model [96]. The obtained microparticles have been shown to slowly desorb antibodies over 30 days in lower dosages. Thus, the systemic administration of free antibodies and analysis of antitumor efficacy indicated pLHMGA microparticles augmented CD8^+^ T cell immune responses, provided long-lasting therapy, and decreased the risk of adverse systemic toxicity in vivo [96].

Inefficient migration and expansion of T cells into solid tumors containing CSCs can be addressed by local delivery of CD8^+^ T cells (or NKG2D-CAR T cells) to the TME of unresectable solid tumors using polymeric alginate scaffolds encapsulated bioactive adjuvants (e.g., IL-15/IL-15Rα fusion protein as IL-15 superagonist) and functionalized with anti- CD3, CD28, and CD137 antibodies, and adhesion peptides (e.g., GFOGER, a collagen-mimetic peptide binding to α2β1 on T cells) [97]. The scaffold-deployed T cells can propagate and stimulate T cells antitumor immunity resulting in the prevention of tumor relapse entirely in a murine model [97]. These findings indicated promising potentials to improve the efficiency of adoptive T cell therapies in an immunosuppressive TME.

As stated above, CSCs impair DCs recruitment and maturation, [4], downregulate the MHC class II and CD80 and CD86 molecules [11], and also impair T cell survival [39]; all interfering with host APC-T cell antitumor activities or neutralizing the immunotherapeutic effects of ACT. Alternative immunotherapy is possible by utilizing the synthetic nanoscale artificial APC (naAPC) (Fig. 3d). Regarding ex vivo products for ACT, magnetic-based naAPC-displaying T cells activating proteins (i.e., MHC-Ig dimer and anti-CD28 antibody) can maximize the TCR clustering as well as T cells expansion and activation to produce antigen-specific T cells [98]. naAPC consists of lipids, magnetic molecules, or polymers that are functionalized with a variety of antigens, ligands (MHC-I Ig G dimer), and T cell-stimulating molecules (i.e., anti-CD28 antibody) [99]. These biomimetic naAPCs have been indicated to stimulate the immune cells and enhance pharmacokinetic properties in vivo [99]. Overall, nano-based delivery systems can be used to deliver existing immunotherapies, augment their efficacy, and improve their potency while reducing toxic side effects. These strategies may be adjusted for simultaneous targeting of CSCs and tumor-supportive immune lineages.

## Conclusion

In summary, our literature surveys the mechanisms through which CSCs and individual immune cells create an immunosuppressive TME-supporting tumor progression and relapse that will help guide the next generation of immunotherapies. Additionally, the CSCs' TAA and CSC-supportive TME can be a target of potent immune agents as monotherapy or in combination with approved drugs to overcome the resistant mechanism in immune evader CSCs. Owing to the shared tumor-initiating and immune evasion properties of CSCs with non-CSCs, CSC's key neoantigens and stemness transcription factor can be targeted by specific immunotherapies. Next, strengthening preclinical studies can accelerate CSC-targeted CAR CIK/T/NK cells in cancer treatment and establish safety in the clinic, ensuring their applications in future clinical cancer treatment. Finally, the nano delivery platforms provide a means to improve the local or systemic delivery of immunotherapies to cancer cells. However, future works and innovations may enable us to adjust nano-immunotherapy as curative approaches to target CSCs with the same efficacy in different tumors.

## Data Availability

Not applicable.

## Abbreviations

ACT: Adoptive cell therapy
APCs: Antigen-presenting cells
CAR: Chimeric antigen receptor
CIKs: Cytokine-induced killer cells
CCL: C–C motif chemokine
CTLA-4: Cytotoxic T lymphocyte antigen-4
DCregs: Immunoregulatory dendritic cells
EMT: Epithelial to mesenchymal transition
MIC A/B: MHC-I polypeptide-related sequence A/B
naAPC: Nanoscale artificial APC
PD-1: Programmed cell death protein-1
SIRPα: Signal-regulatory protein α
TAMs: Tumor-associated macrophages
TME: Tumor microenvironment
Tregs: Regulatory T cells
